# Biomimetic Engineering Preparation of High Mechanical and Flame Retardant Elastomers by Introducing Sacrificial Bonds in Covalently Cross-Linked Chloroprene Rubber

**DOI:** 10.3390/polym15163367

**Published:** 2023-08-10

**Authors:** Jianliang Jiang, Junxue Zhai, Yiqun Zhang, Yakai Feng

**Affiliations:** 1 School of Chemical Engineering and Technology, Tianjin University, Yaguan Road 135, Tianjin 300350, China; 2 China (Yangzhou) Material Handling Tech-Engineering Ltd., Hongyang Road 66, Yangzhou 225000, China; 3 Key Laboratory of Rubber-Plastics of Ministry of Education (QUST), School of Polymer Science & Engineering, Qingdao University of Science & Technology, Zhengzhou Road 5, Qingdao 266042, China; 4 Frontiers Science Center for Synthetic Biology, Tianjin University, Yaguan Road 135, Tianjin 300350, China; 5 Key Laboratory of Systems Bioengineering (MOE), Tianjin University, Yaguan Road 135, Tianjin 300350, China

**Keywords:** mechanical, flame retardancy, sacrificial bond, chloroprene rubber

## Abstract

Designing and preparing chloroprene rubber (CR) with robust mechanical and excellent flame retardancy performance are challenging. In this work, a biomimetic design for high mechanical and flame-retardant CR by synchronous introducing of sacrificial bond (disulfide) crosslinked networks into the chemically crosslinked network is developed based on two new types of vulcanization reactions. Under the catalysis of $Mg{\left(OH\right)}_{2}$, the dynamic bond cross-linked network is formed by the reaction between the amino group of cystamine dihydrochloride (CA) and the allylic chlorine group of CR, while the covalently crosslinked network is synchronously formed by two types of nucleophilic substitution reactions in series between $Mg{\left(OH\right)}_{2}\;$ and CR. The disulfide bonds serve as sacrificial bonds that preferentially rupture prior to the covalent network, dissipating energy and facilitating rubber chain orientation, so a CA-0.5 sample (CR/CA(0.5 wt%)/$Mg{\left(OH\right)}_{2}$ (10 wt%) with dual crosslinked networks exhibits excellent mechanical performance, and the tensile strength and elongation at the break of CA-0.5 are 1.41 times and 1.17 times greater than those of the CR-0 sample with covalently crosslinked networks, respectively. Moreover, the addition of $Mg{\left(OH\right)}_{2}$ significantly improves the flame retardancy of CR.

## 1. Introduction

Hydrogel-like bio-tissues, such as tendon [1], silk [2], and byssus [3], exhibit fascinating mechanical properties. It has been widely accepted that the high mechanical abilities of biological materials originate from the dynamics of sacrificial bonding systems: the rupture of sacrificial bonds and the release of hidden lengths dissipate a large amount of energy [4,5]. For example, hydrogen bonds and metal–ligand coordination bonds were found to serve as sacrificial bonds in spider silk proteins [6,7] and mussel byssal threads [3,8], respectively. Inspired by the sacrificial bonding structures in biological materials, various non-covalent bonds, as well as covalent ones, as sacrificial bonds have been engineered into artificial hydrogels and elastomers.

Gong et al. performed the pioneering work on the construction of double-network hydrogels by two-step sequential free radical polymerization with covalent sacrificial bonds [9,10]. The first network is tightly crosslinked, while the second network is loosely crosslinked. Therefore, the first brittle network serves as sacrificial bonds, breaking into small clusters to efficiently disperse the stress around the crack tip into the surrounding damage zone, while the second ductile polymer chains act as hidden lengths, which extend extensively to sustain large deformation. However, the rupture of the sacrificial covalent bonds causes permanent damage to the materials. To enable recoverable energy-dissipating mechanisms, the introduction of reversible bonds, such as hydrogen-bonding, metal−ligand, and ionic interactions [11] as sacrificial bonds in the hydrogel matrix, has been extensively studied [12]. Guo et al. prepared hydrogels with physical crosslinks comprising 2-ureido-4[1H]-pyrimidinone (UPy) hydrogen-bonding units within the backbone of segmented amphiphilic macromolecules; due to the microphase separated network, the hydrogels exhibited high strength and resilience upon deformation [13]. Zhao et al. prepared inter-network ionically crosslinked hybrid double network (DN) hydrogels from poly(sodium 2-acrylamide-2-methylpropanesulfonate) (PNaAMPS) as the first network and polyacrylamide (PAM) as the second network. Hybrid DN hydrogels with enhanced strength were obtained near the ion-balance point [14]. Sakai et al. synthesized a composite polyvinyl alcohol/poly(acrylic acid)/silicone double network hydrogel by the introduction of sacrificial bonds and evaluated its mechanical performance [15].

The sacrificial strategy has also been used to toughen elastomers [16,17,18]. Liu et al. proposed a novel strategy to engineer a multinetwork by incorporating weaker sacrificial hydrogen bonds and stronger Zn-based units into a chemically crosslinked cis-1,4-polyisoprene network through a three-step independent reaction process [19]. Upon loading, the hydrogen bonds and Zn-based bonds break in sequence and recombine to dissipate energy, significantly improving the tensile modulus and fracture toughness. Tang et al. obtained a butadiene–styrene–vinylpyridine rubber (VPR) with improved tensile strength and toughness by incorporating dynamical Zn−pyridine motifs into a chemically crosslinked architecture network [20]. Zhu et al. prepared a doubly reversible crosslinked styrene-butadiene rubber (SBR) through a two-step reaction [21]. First, commercial SBR was epoxidized by controlled phase-transfer catalysis and then covalently crosslinked with HA via the reaction between epoxy and phenolic hydroxyl groups to form elastomers. The integration of dynamical hydrogen bonding endowed SBR elastomer with significantly improved strength and toughness. You et al. obtained transparent interlocked networks composed of the first network of Diels–Alder bonds crosslinked with SBR and a second network of imine bonds crosslinked with polyethylenimine (PEI) [22]. Qiu et al. developed lignin/carbon black/nitrile rubber elastomers with high performance by constructing a dual-crosslinking network consisting of covalent bonds and dynamic Zn-coordination sacrificial bonds [23]. The multilevel energy consumption mechanisms of the interlocking architecture and the inter-macromolecular ionic interactions promote the simultaneous improvement of the strength, toughness and creep resistance. However, the processes of sacrificial strategies to prepare engineered rubber elastomers are cumbersome. Engineering sacrificial construction into elastomers simply has been widely pursued by scientists.

The preparation of chloroprene rubber (CR) with excellent mechanical properties and flame retardancy using traditional rubber processing equipment could greatly expand its application fields. The current research mostly focuses on filler-type flame retardants [24,25], ultimately leading to compromised mechanical and flame retardancy [26]. In our previous study, hydrogen-bonding sacrificial bonds were introduced in CR rubber, achieving a combination of improved mechanical properties and flame retardancy [27]. In this work, we developed a simple method for preparing CR vulcanizates with dual crosslinked networks based on two new types of vulcanization reactions, in which disulfide bonds, serving as sacrificial bonds, break prior to the covalent bonds, dissipate energy and facilitate CR rubber chain orientation, achieving high mechanical properties and good flame retardancy.

## 2. Experiment

### 2.1. Materials

Chloroprene rubber (CR3221) was supplied by Chongqing Changshou Chemical Co., Ltd., Chongqing, China. Aluminum hydroxide ($Al{\left(OH\right)}_{3}$), magnesium hydroxide ($Mg{\left(OH\right)}_{2}$), 4,4′-dithiodianiline (DTA) and cystamine dihydrochloride (CA) were supplied by Shanghai Yien Chemical Technology Co., Ltd., Shanghai, China. Carbon black N550 (CB) and pentaerythritol tetrakys 3-(3,5-ditert-butyl-4-hydroxyphenyl) propionate (antioxidant 1010) were provided by Energy Chemical Technology (Shanghai) Co., Ltd., Shanghai, China. All of these reagents were used as received.

### 2.2. Preparation of CR Vulcanizates

All components were oven-dried at 45 °C for 24 h before use. The samples were prepared at room temperature and less than 50% relative humidity. In a typical procedure, CA, $Mg{\left(OH\right)}_{2}$, and antioxidant 1010 were successively compounded with CR3221 on a double-roll mill (X(S)K-160) within 15 min. After mixing, the compounds were subjected to compression at a set temperature for the optimum curing time determined by a U-CAN UR-2030 (Taiwan) vulcameter.

The blend designations and compositions are shown in Table 1. CA1-x and DTA1-x refer to the addition of different masses of $Mg{\left(OH\right)}_{2}$ to the CR mixture, such as CA1-15, which refers to the addition of 15 wt% of $Mg{\left(OH\right)}_{2}$ to the CA-1 compound.

### 2.3. Measurement and Characterization

The vulcanization characteristics were tested according to ASTM D2084-2001 using a Moving Die Rhometer GT-M2000-A (Gaotie Test Instruments Factory, Taiwan, China) at 160 °C for 30 min. The tensile properties of the samples were tested according to GB/T 528-2009 using a GT-AI-7000-S stretching machine (Gaotie Test Instruments Factory, Taiwan, China) with a speed of 500 mm/min at room temperature. The average value of five individual samples was recorded for each sample. The average value of three individual samples was recorded for each sample. ATR-FTIR spectra were recorded on a VERTEX70 spectrometer (Bruker, Germany). Samples were characterized by signal averaging 32 scans at a resolution of 4 cm^−1^ in the wavenumber range of 500–4000 cm^−1^. The thermal degradation behaviors of the composites were studied by a thermal gravimetric analyzer (TGA, TG209, NETZSCH, Instruments, Selb, Germany) at a heating rate of 10 °C/min under a nitrogen atmosphere. Differential scanning calorimetry (DSC) analysis was carried out to study the crosslinking behaviors of samples using TA equipment (SDTQ20, New Castle, DE, USA). The samples were heated to 250 °C at a rate of 10 °C/min under N_2_ flow. The limiting oxygen index of the samples were tested by an HC-2 oxygen index tester according to the method of GB/T 2406.2-2009 [27].

## 3. Results and Discussion

### 3.1. The Vulcanization Characteristics of CR

**Scheme 1:**
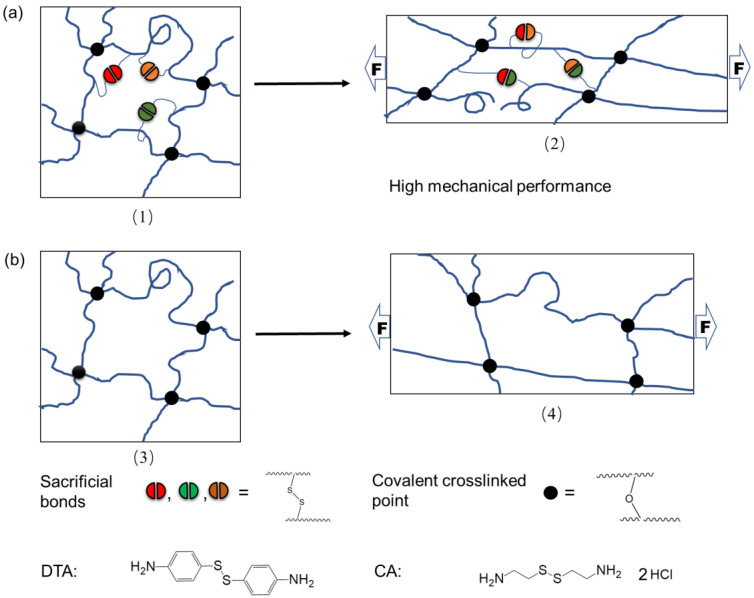
Schematic diagram of the tensile mechanism of CR vulcanizate. (**a**) Double network CR vulcanizate with sacrificial bond crosslinks; (**b**) single network CR vulcanizate with covalent bond crosslinks.

The vulcanization of CR is generally achieved by the reaction between the allylic chlorine groups (C=C-C-Cl) of CR and different curing agents (such as zinc oxide and magnesium oxide) to form different covalent networks [28]. It has been reported in the literature that allylic chlorine groups of small compounds can react with amino groups under mild conditions [29]. So it is inferred that allylic chlorine groups on CR polymer chains can also react with amino groups during vulcanization. To simplify the introduction of disulfide bonds in CR vulcanizates, a new type of vulcanizing agent was selected, which contains two terminal amino groups and a disulfide bond. Considering the price and accessibility, two vulcanizing agents were ultimately selected, namely CA and DTA (Scheme 1). An appropriate amount of base needs to be added to absorb the acid generated by the reaction, and considering the improvement of CR flame retardancy, a strategy of “killing two birds with one stone” was proposed, using traditional flame retardants [30], $\;Mg{\left(OH\right)}_{2}$ or $Al{\left(OH\right)}_{3}$, as acid absorbers.

**Figure 1:**
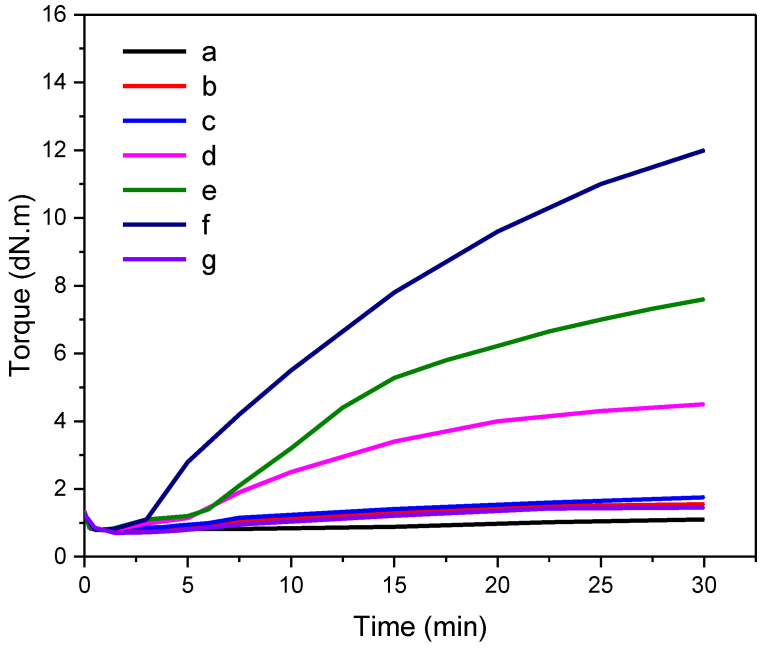
The vulcanization curves of CR with different compounds at 160 °C. (a) CR; (b) CR/CA (1 wt%); (c) CR-2; (d) CR-1; (e) CA-1; (f) DTA-1; (g) CR/DTA(1 wt%).

**Figure 2:**
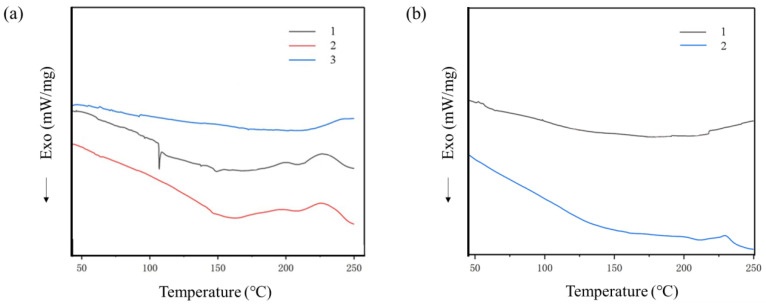
(**a**) DSC curves of different CR/CA mixtures during heating: (1) CR-2; (2) CA-1; (3) CR/CA (1 wt%). (**b**) DSC curves of different CR/DTA mixtures during heating: (1) CR/DTA (1 wt%); (2) DTA-1. The arrow means exothermic.

**Figure 3:**
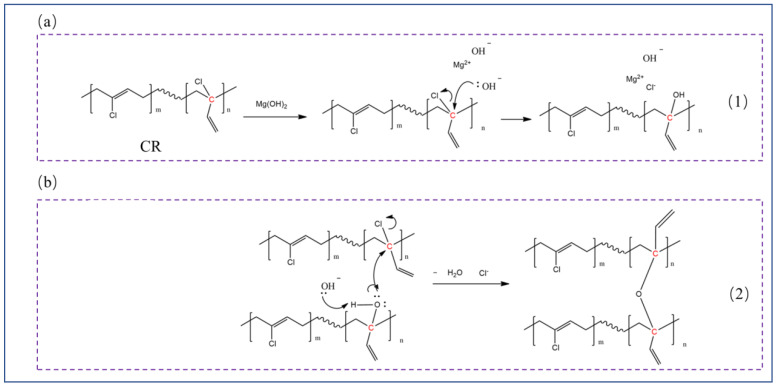
Possible reactions between CR and Mg(OH)_2_. (**a**) Reaction 1, the nucleophilic substitution reaction of allylic chlorine groups by ${\mathrm{OH}}^{-}$ groups; (**b**) Reaction 2, a nucleophilic substitution reaction of allylic chlorine groups by the hydroxyl groups under catalysis of $Mg{\left(OH\right)}_{2}$.

First, the effect of the inorganic base on the vulcanization of CR was investigated. As shown in Figure 1, the addition of $Al{\left(OH\right)}_{3}$ exhibited almost no vulcanization, but surprisingly, $Mg{\left(OH\right)}_{2}$ resulted in a significant vulcanization reaction of CR at 160 °C. $Mg{\left(OH\right)}_{2}$ has been applied as a vulcanizing agent for CR, so we proposed a hypothesis: $Mg{\left(OH\right)}_{2}$ decomposes into magnesium oxide at high temperatures, causing the crosslinking reaction of CR. According to the literature, the thermal decomposition temperature of $Mg{\left(OH\right)}_{2}$ is about 377 °C [31], consistent with the TGA experimental results, but no significant thermal weight loss was found around 160 °C. These experimental results indicated that our hypothesis was incorrect because $Mg{\left(OH\right)}_{2}$ cannot decompose at the vulcanization temperature. As shown in Figure 2, the DSC result confirmed that CR did react with $Mg{\left(OH\right)}_{2}\;$ to release heat at temperatures ranging from 100 to 200 °C. Therefore, we proposed a possible mechanism for the vulcanization reaction of $Mg{\left(OH\right)}_{2}\;$ and CR in Figure 3. This mechanism involves two-step cascade reactions: Reaction 1, the nucleophilic substitution reaction of allylic chlorine groups by ${OH}^{-}$ groups; and Reaction 2, a nucleophilic substitution reaction of allylic chlorine groups by the hydroxyl groups under catalysis of $Mg{\left(OH\right)}_{2}$, which is the so-called Williamson reaction. As shown in Figure 1d, the maximum torque of the CR-2 sample gradually increases with time, indicating that the apparent reaction rate of Reaction 1 is lower than that of Reaction 2. The weight content of the monomers containing a C=C-C-Cl group in CR is about 1.5%. It can be calculated that the molar ratio of ${\mathrm{OH}}^{-}$ to the C=C-C-Cl group (=10.1) is far greater than 1 when the content of $Mg{\left(OH\right)}_{2}$ is 10 wt%. If the apparent rate of Reaction 1 is greater than that of Reaction 2, all the C=C-C-Cl groups of CR will be converted into C=C-C-OH groups, and the vulcanization curve of CR-2 should not exhibit the characteristic of a crosslinking reaction, in contrast to the experimental results. Therefore, the apparent reaction rate of Reaction 1 is that of Reaction 2. The dissolution test showed that CR-2 vulcanizate could not be completely dissolved in chloroform but formed a gel, indicating that a covalent bond crosslinking network was formed. Due to the weak alkalinity of $Al{\left(OH\right)}_{3}$, Reaction 1 and Reaction 2 are less likely to occur at vulcanization temperatures, so the CR-1 vulcanization curve did not exhibit the occurrence of crosslinking reactions (Figure 1c).

Subsequently, the effect of CA containing aliphatic amino groups on the vulcanization characteristics of CR rubber was also studied. As shown in Figure 1a,b, it can be seen that the torque of CR/CA (1 wt%) without a base does not increase with time at the curing temperature, indicating that the crosslinking reaction can hardly take place. A possible reason is that the amino group of the vulcanizing agent cysteamine hydrochloride exists in the form of a positive ion (${R-NH}_{3}^{+}$), so the N atom cannot provide lone pair electrons, resulting in the inability of crosslinking reaction to occur. In comparison, when CA and $Mg{\left(OH\right)}_{2}$ were added simultaneously, as shown in Figure 1f, the vulcanization reaction of CA-1 was obvious, and the maximum torque of CA-1 was 1.7 times higher than that of CR-1 (Figure 1d). It can be inferred that the CA-1 system formed not only a covalent crosslinking network similar to that of the CR-1 vulcanizate but also a new crosslinking network between CR and CA. The results of DSC experiments (Figure 2) showed that, when CA (1 wt%) and $Mg{\left(OH\right)}_{2}\;$(10 wt%) were added simultaneously to CR, CA-1 exhibited a wide and obvious exothermic peak between 120–180 °C (Figure 2a), indicating that CA did indeed undergo a vulcanization reaction with CR. Due to the small amount of CA added (the molar ratio of $Mg{\left(OH\right)}_{2}$ to CA is 38.5), it can be inferred that $Mg{\left(OH\right)}_{2}\;$ first reacts with HCl in cystamine dihydrochloride, releasing the unprotonated amino groups, which subsequently reacted with the allylic chlorine groups of CR under the catalysis of $Mg{\left(OH\right)}_{2}$.

**Figure 4:**
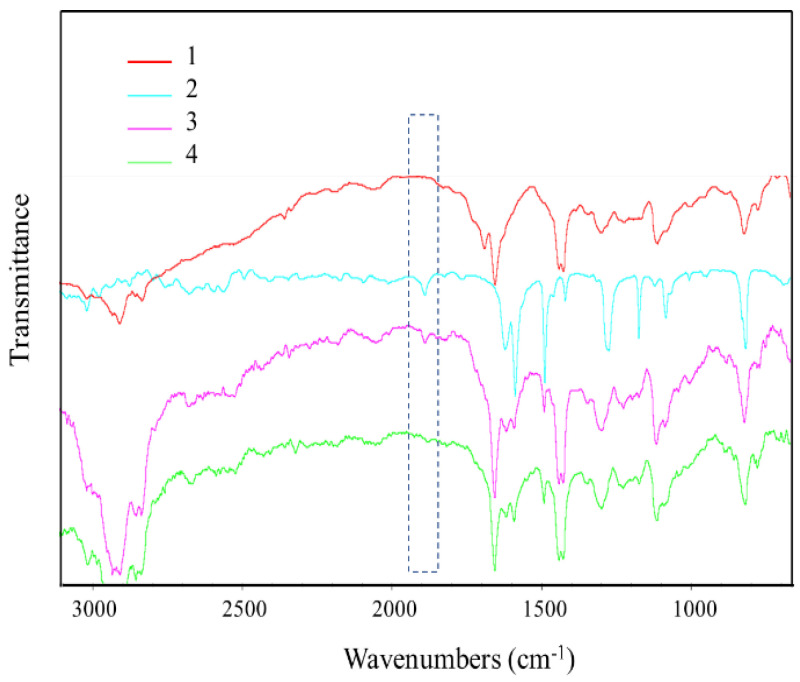
FTIR spectra of different compounds. (1) CR; (2) DTA; (3) CR/DTA (3 wt%)/Mg(OH)_2_ (10 wt%) without vulcanization; (4) CR/DTA (3 wt%)/Mg(OH)_2_ (10 wt%)− after vulcanization.

Considering that the chemical environment of the primary amino group leads to differences in the reactivity of the amino group, as a comparison sample, the effect of DTA containing aromatic amino groups on the vulcanization characteristics of CR rubber was also determined. As shown in Figure 1g, when DTA is added alone, CR/1 wt% DTA sample also cannot be vulcanized. A possible reason is that, although the amino groups of DTA are not protonated (the amino groups of CA dihydrochloride have been protonated), the lone pair electrons of the N atom have conjugated with the π bond of the benzene ring, reducing the nucleophilic ability of the N atom, also causing the CR/DTA (1 wt%) sample to not be able to be vulcanized. Compared with Figure 1f,g, the adding of $Mg{\left(OH\right)}_{2}$ exhibits obvious vulcanization, and the maximum torque of DTA-1 was 2.7 times higher than that of CR-1, indicating that $Mg{\left(OH\right)}_{2}$ promoted the vulcanization reaction of -NH_2_ of DTA with the allylic chlorine groups of CR. The catalysis of $Mg{\left(OH\right)}_{2}$ on the vulcanization in the DTA-1 system is consistent with that in the CA-1 system. As shown in Figure 2b, it can be observed that, when 1 wt% DTA was added separately to CR, no obvious heating peak was observed during the heating process, indicating that the vulcanization reaction of CR/DTA (1 wt%) cannot take place. When DTA (1 wt%) and $Mg{\left(OH\right)}_{2}$ (10 wt%) were simultaneously added to CR, the obtained sample exhibited a wide and obvious exothermic peak between 120 and 180 °C (Figure 2b), indicating that the vulcanization reaction can take place under the catalysis of $Mg{\left(OH\right)}_{2}$. Finally, the vulcanization reaction was characterized by FTIR. Due to the hydrogen bonding, the characteristic absorption band of -NH_2_ and ${\mathrm{OH}}^{-}$ is located at 3000–3500 cm^−1^ [32], while the absorption band of =N-H bond generated by vulcanization reactions also is located at 3000–3500 cm^−1^. Therefore, it is not suitable to judge the occurrence of the vulcanization reaction based on the characteristic absorption band intensity at 3000–3500 cm^−1^. As shown in Figure 4, the band located at 1892 cm^−1^ is used as the characteristic absorption peak to determine the occurrence of the reaction, as DTA has absorption at 1892 cm^−1^, while CR rubber has no absorption at 1892 cm^−1^. The band can be observed at 1892 cm^−1^ for CR/DTA before vulcanization, and it disappeared after vulcanization; therefore, it can be inferred that a reaction did take lace between DTA and CR.

**Figure 5:**
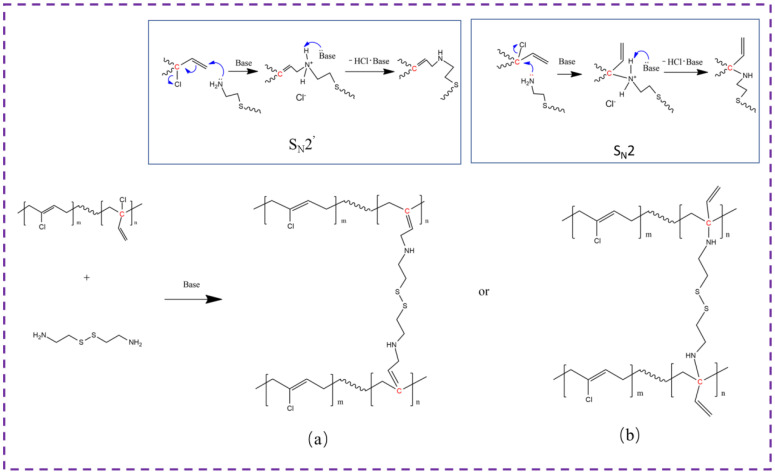
Possible reactions between CR and CA under the catalysis of Mg(OH)_2_. (**a**) nucleophilic substitution reaction via ${SN}_{2}^{\prime }$ reaction mechanism; (**b**) nucleophilic substitution reaction via ${SN}_{2}^{}$ reaction mechanism.

Based on the above experimental results, taking CR/CA as an example, a possible reaction mechanism between CR and CA under the catalysis of $Mg{\left(OH\right)}_{2}$ is proposed in Figure 5. First, CA is neutralized by $Mg{\left(OH\right)}_{2}$, releasing free cystamine; then, a nucleophilic substitution reaction of the ${-NH}_{2}$ group on the C=C-C-Cl group takes place. Because both C^1^ and C^3^ atoms in the C^1^=C^2^-C^3^-Cl group are positive, they can be attacked by the N atom under the catalysis of $Mg{\left(OH\right)}_{2}$. A positive intermediate is formed when the ${\mathrm{Cl}}^{-}$ is removed, which is dehydrated under the catalysis of $Mg{\left(OH\right)}_{2}\;$ to form covalent crosslinked CR with a disulfide bond in each crosslinker. Considering the steric hindrance effect, the ${SN}_{2}^{\prime }$ reaction preferentially take place. Combined with the crosslinking effect of $CR/Mg{\left(OH\right)}_{2}$, as shown in Figure 5, CA-1 vulcanizate will form a dual network structure, namely a covalent network and a dynamic network.

### 3.2. Vulcanization Performance of CR with Dual Networks

**Figure 6:**
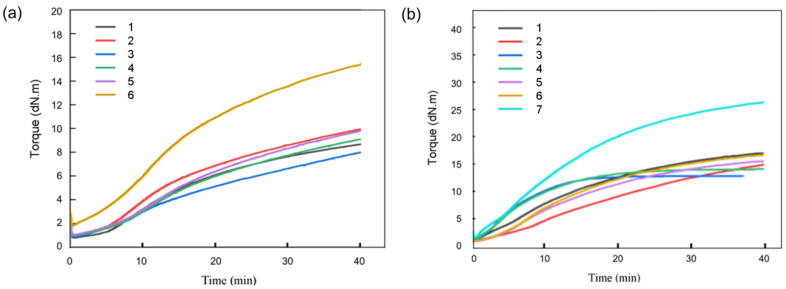
(**a**) Vulcanization curves of different mixtures containing CA at 160 °C: (1) CA-1; (2) CA-0.5; (3) CA-2; (4) CA-1-15; (5) CA-1-20; (6) CA 1-10-20. (**b**) Vulcanization curves of different mixtures containing DTA at 160 °C: (1) DTA-1; (2) DTA-0.5; (3) DTA-3; (4) DTA-2; (5) DTA-1-15; (6) DTA-1-20; (7) DTA 1-10-20.

Following the understanding of the vulcanization mechanisms of CR/CA/$Mg{\left(OH\right)}_{2}$, experiments were conducted to optimize the vulcanization performance of CR/CA/$Mg{\left(OH\right)}_{2}$. First, the effect of $Mg{\left(OH\right)}_{2}$ content on vulcanization performance was investigated. The apparent vulcanization rate (estimated by the slope of the vulcanization curve) is the sum of two reaction rates, namely the reaction rate for forming a covalent crosslinking network (Figure 3) and the reaction rate for forming a dynamic crosslinking network (Figure 5). As shown in Figure 6a, the vulcanization rates of the three samples (1 and 4–5 curves) were consistent within 15 min; after 15 min, the vulcanization rate and maximum torque both increased with the increase in $Mg{\left(OH\right)}_{2}$ content because the reaction rate between CA and CR catalyzed by $Mg{\left(OH\right)}_{2}$ was much higher than that between $Mg{\left(OH\right)}_{2}$ and CR. Therefore, the apparent vulcanization reaction rate in the early stage was mainly the crosslinking reaction rate between CA and CR. After 15 min, the vulcanization reaction consumed all CA raw materials; therefore, the apparent crosslinking reaction rate was mainly the reaction rate between CR and $Mg{\left(OH\right)}_{2}$. Second, the effect of CA content on the vulcanization performance of CR/CA is shown in Figure 6a (1–3 curves). The vulcanization rate and maximum torque gradually decreased with the increase in CA content. The number of monomers containing C=C-C-Cl groups in the CR polymer chain is small [33]. When the molar ratio of CA/C=C-C-Cl is greater than 0.5, only one amino group of some CA molecules will participate in the reaction, thus reducing the crosslinking density of the vulcanizate. The torque of the sample increased slowly with time, indicating that the vulcanization rate between $Mg{\left(OH\right)}_{2}$ and CR was very slow, and the covalent crosslinked network density can be easily tuned by controlling the vulcanization time. Finally, the effect of carbon black on vulcanization was also determined. Comparing the vulcanization curves in Figure 6a (1 and 6 curves), it can be observed that the addition of carbon black significantly increased the initial and maximum torque of CR vulcanizate. The maximum torque of CA1-10-20 was 1.77 times higher than that of CA-1. Compared to CA-1, the apparent vulcanization rate of CA1-10-20 slightly increased. 

In comparison, the vulcanization performance of CR/DTA was also assessed. First, the influence of DTA content on vulcanization performance is shown in Figure 6b (1–4 curves). In the initial stage, the crosslinking rate increased with the increase in DTA content, while the maximum torque of the DTA-1 was the highest. Second, the effect of $Mg{\left(OH\right)}_{2}$ content on the vulcanization performance of CR/DTA was also investigated. As shown in Figure 6b (1 and 5–6 curves), in the later stage, the vulcanization rates of CR-1-x samples were basically consistent, which might be due to the crosslinking rate of CR/DTA being basically the same as that of CR/$Mg{\left(OH\right)}_{2}$. In addition, the maximum torque of DTA-1-10 vulcanizate was the highest. Finally, the effect of carbon black on vulcanization is shown in Figure 6b (1 and 6 curves), and it can be seen that the vulcanization rate of DTA1-10-20 was significantly higher than that of DTA-1 in the initial stage, and the maximum torque of DTA1-10-20 was 1.53 times higher than that of DTA-1.

### 3.3. Tensile Properties of CR Vulcanizate

**Figure 7:**
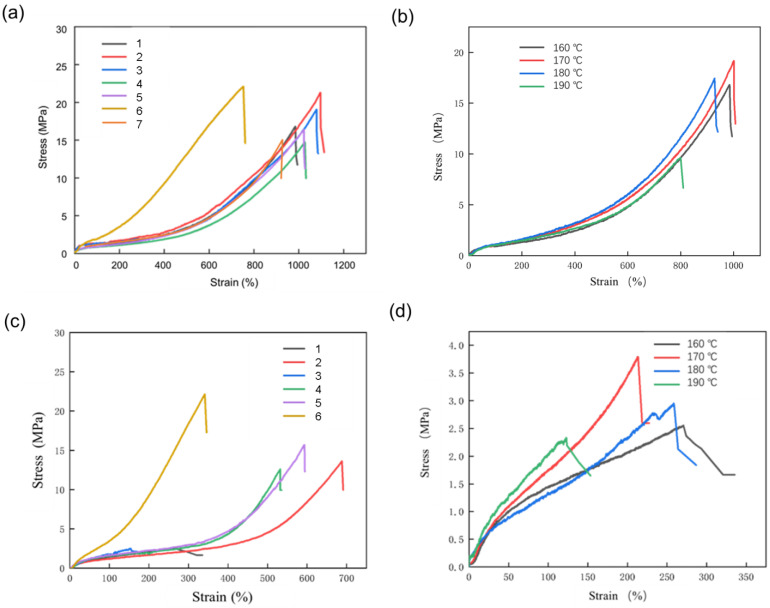
(**a**) The stress and strain curves of different CR/CA blends at 160 °C: (1) CA-1; (2) CA-0.5; (3) CA-2; (4) CA-1-15 wt%; (5) CA-1-20; (6) CA1-10-20; (7) CR-0. (**b**) Tensile curves of CA-1 at different vulcanization temperatures. (**c**) The stress and strain curves of different CR/DTA blends: (1) DTA-1; (2) DTA-0.5; (3) DTA-2; (4) DTA-1-15; (5) DTA-1-20; (6) DTA1-10-20. (**d**) Tensile curves of DTA-1 at different vulcanization temperatures.

Mechanical properties are fundamental properties for engineering materials. A series of experiments were conducted to optimize the mechanical properties of CR/CA vulcanizates. Before carrying out the comparative study of the mechanical properties of CR vulcanizates mentioned above, a covalently crosslinked CR-0 was prepared as a benchmark. As shown in Figure 7a, the tensile strength and elongation at breaks of CR-0 vulcanizate crosslinked by zinc oxide were 15.4 MPa and 928%, respectively. The effect of CA content on the mechanical properties of CR/CA vulcanizate was investigated. As shown in Figure 7a (1–3 curves), it can be observed that the tensile strength gradually decreased with the increase in CA content. The tensile strength and elongation at the break of CA-0.5 were both the highest, reaching 21.7 MPa and 1090%, respectively. It is gratifying that CA-0.5 vulcanizate has higher tensile strength, as well as much greater elongation, at breaks than the control samples (CR-0). The good mechanical properties of CA-0.5 are attributed to the formation of a dual crosslinked network structure, in which the disulfide crosslinking bonds serve as sacrificial bonds, which continuously break and regenerate during the stretching process, dissipating energy and promoting the orientation of the rubber chain. The effect of $Mg{\left(OH\right)}_{2}\;$ content on the mechanical properties of CR/CA vulcanizate was carried out. As shown in Figure 7a (1 and 4, 5 curves), the tensile strength of CR/CA vulcanizate decreased, and the elongation at breaks increased slightly with the increase in $Mg{\left(OH\right)}_{2}\;$ content. This phenomenon can be attributed to the existence of an optimal balance between the covalent crosslinking density and the dynamic crosslinking density within the dual network system. In the CA-x system, the allylic chlorine group of CR participated in the reaction to form both covalent and dynamic crosslinking bonds. As the number of allylic chlorine groups in CR is small, the molar ratio of the covalent crosslinking bonds to the dynamic crosslinking bonds will increase with the increase in $Mg{\left(OH\right)}_{2}$ content, which deviates from the optimal ratio, leading to the decline in the tensile strength of CR/CA vulcanizates. The effect of vulcanization temperature on the mechanical properties of CA-1 vulcanizate was carried out. As shown in Figure 7b, CA-1 samples obtained at a vulcanization temperature of 170 °C, exhibited maximum tensile strength, as well as maximum elongation at breaks. When the vulcanization temperature is higher than 170 °C, the tensile strength and elongation at the breaks of the CA-1 vulcanizate slightly decreased, which may be due to side reactions leading to uneven crosslinking networks.

In comparison, the mechanical properties of CR/DTA vulcanizates were also determined. As shown in Figure 7c, compared to DTA-1 and DTA-2, DTA-0.5 vulcanizate exhibited maximum tensile strength (13.3 MPa) and maximum elongation at breaks (682%), because more crosslinking bonds containing disulfide bonds formed in the DTA-0.5. When the DTA content was in excess (the molar ratio of DTA/C=C-C-Cl was greater than 0.5), the number of effective crosslinks containing disulfide bonds formed in CR vulcanizate decreased. However, DTA-0.5 vulcanizate has lower tensile strength, as well as much lower elongation at breaks than CA-0.5 vulcanizate and the control sample (CR-0). On the one hand, there may be an undeniable side reaction between DTA and CR [34], leading to a decrease in effective crosslinking bonds containing disulfide bonds. On the other hand, the strength of disulfide bonds in CA is greater than that in DTA, while the exchange ability of aliphatic disulfide bonds (CA) is lower than that of aromatic disulfide bonds [35]. The effect of $Mg{\left(OH\right)}_{2}$ content on the mechanical properties of CR/DTA vulcanizates was also assessed. As shown in Figure 7c (1, 4 and 5 curves), the tensile strength and elongation at breaks of CR/DTA vulcanizate gradually increased with the increase in $Mg{\left(OH\right)}_{2}$ content. A possible reason is that the addition of $Mg{\left(OH\right)}_{2}$ promoted both the reaction of CR/DTA and the reaction of CR/$Mg{\left(OH\right)}_{2}$, and DTA-1-20 has higher covalent crosslinking density and higher dynamic crosslinking density than DTA-1 and DTA-1-15. The mechanical properties of CR/DTA vulcanizate reinforced with carbon black were also investigated. As shown in Figure 7c (2 and 7 curves), the addition of carbon black significantly improved the tensile strength of CR vulcanizate, increasing from 13.3 MPa to 22.1 MPa, but it sacrificed the elongation at breaks, decreasing from 682% to 340%. The modulus of DTA1-10-20 vulcanizate was also significantly improved. The effect of vulcanization temperature on the mechanical properties of DTA-1 was investigated. As shown in Figure 7d, the DTA-1 sample synthesized under a vulcanization temperature of 170 °C exhibited the highest tensile strength among all samples, while the DTA-1 sample prepared at a vulcanization temperature of 190 °C exhibited a significant decrease in both tensile strength and elongation at breaks.

Comparing the mechanical performances of CR/CA, CR/DTA and CR/ZnO vulcanizates, it can be seen that CA-0.5 has the best mechanical properties. Compared to CR-0 vulcanizate with covalently crosslinking networks, the tensile strength and elongation at breaks of CA-0.5 were simultaneously improved. The schematic diagram is shown in Scheme 1. The superior mechanical properties observed in the CA-0.5 vulcanizate are primarily attributed to the establishment of a dual network structure, wherein the dynamic crosslinks featuring disulfide bonds act as sacrificial bonds [20], continuously breaking and restructuring during the stretching process to optimize the network of CR vulcanizate, dissipating a large amount of energy and achieving excellent mechanical properties.

### 3.4. Thermal Analysis of CR/CA Vulcanizates

**Figure 8:**
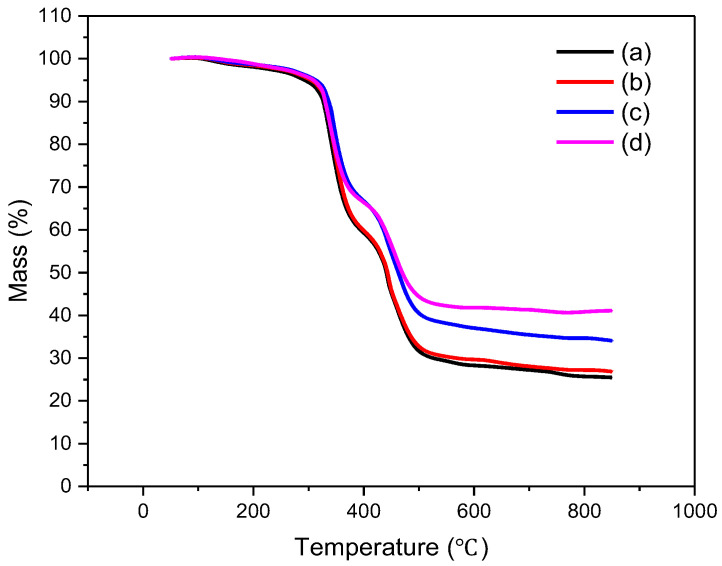
Thermogravimetric curves of different compounds: (a) CA-1; (b) CA-0.5; (c) CA-1-20; (d) CA1-10-20.

The effect of different additives on the heat resistance of CR/CA vulcanizate was determined using a thermogravimetric analyzer. As shown in Figure 8, the thermal weight loss curves of all four samples exhibited the typical two-stage rapid weight loss characteristics of polymers. The initial temperature of thermal weight loss (T_5%_) for the four samples were 292.3, 304.2, 312.8, and 308.6 °C, respectively, indicating good thermal stability of CR/CA. The mass of the four samples slowly decreased at 360~430 °C, and the final residual weights of the four samples were 25.5, 26.8, 34.1 and 41.1 wt%, respectively. As shown in Figure 8a,b, in contrast to CA-1, the final residual weight of CA-0.5 exhibited an increase from 25.5 to 26.8 wt%, signifying the adverse role of CA in reducing the thermal weight loss of CR when exposed to elevated temperatures. As shown in Figure 8a,c, compared to CA-1 vulcanizate, the content of $Mg{\left(OH\right)}_{2}\;$ in the CA-1-20 sample increased by 10 wt%, while the final residual weight of CA-1-20 sample increased by 8.6 wt%. $Mg{\left(OH\right)}_{2}\;$ is a classic additive inorganic flame retardant that decomposes into water and magnesium oxide at 350 °C and absorbs heat [25]. The water vapor generated by decomposition of $Mg{\left(OH\right)}_{2}$ will evaporate from the system into the surrounding environment. Assuming that all the $Mg{\left(OH\right)}_{2}\;$ is converted into magnesium oxide, the final residual weight should theoretically increase by 6.2 wt%, which is smaller than the actual increase in residual weight (8.6 wt%). Therefore, it can be inferred that $Mg{\left(OH\right)}_{2}$ can alleviate the thermal weight loss of CR vulcanizate. As shown in Figure 8a,d, compared to CA-1, the T_5%_ of CA1-1-20 vulcanizate slightly increased, while the final residual weight sharply increased by 16.1 wt%. Assuming that carbon black is stable and does not lose weight during the heating process, the final residual weight should theoretically increase by 17.8 wt%, which is greater than the actual value. Therefore, carbon black has a slight promoting effect on the thermal weight loss of CR/CA vulcanizate.

### 3.5. Flame Retardancy of CR Vulcanizates

**Figure 9:**
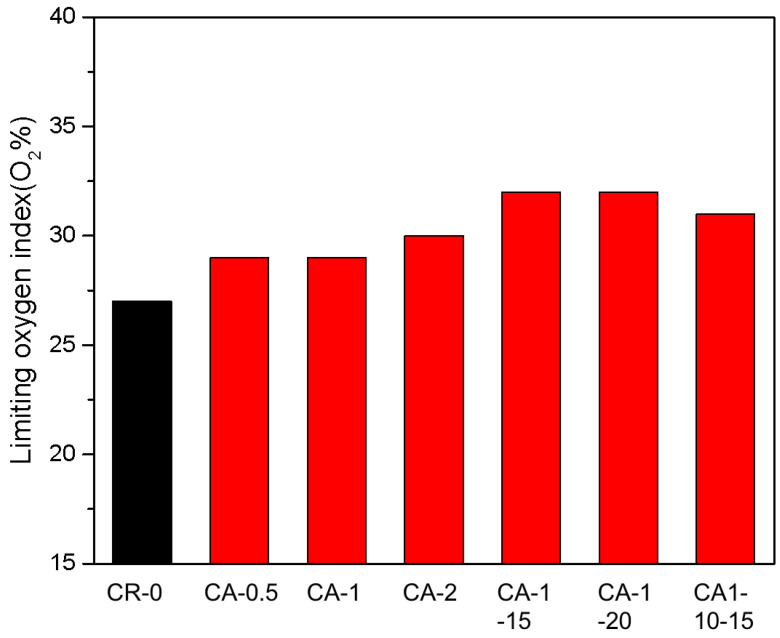
Limiting oxygen indices of different CR vulcanizates.

The halogen atoms present along the polymer chain have the ability to capture free radicals produced during the combustion process. The CR polymer chain contains a large number of C–Cl bonds, endowing the CR vulcanizate with flame-retardant properties [36]. To enhance the flame-retardant properties of CR, it is typically essential to incorporate supplementary flame retardants, which facilitate the suitability of CR vulcanizates for applications that demand high levels of flame retardancy. The vulcanization experiment demonstrated that, apart from serving as a vulcanizing agent, $Mg{\left(OH\right)}_{2}$ also functioned as a catalyst for the reaction between amino groups and allylic chlorine. When the vulcanization formula was initially designed, the role of $Mg{\left(OH\right)}_{2}$ as a flame retardant was also considered. Therefore, flame retardant performance tests of the CR/CA system were carried out. As shown in Figure 9, all six samples exhibited higher LOI values than the CR-0 sample in the absence of $Mg{\left(OH\right)}_{2}$. The flame retardancy of $Mg{\left(OH\right)}_{2}$ is attributed to the endothermic effect and the dilution oxygen effect of water vapor generated during thermal decomposition. It can be also seen that the LOI of CA-x vulcanizate gradually increased with the increase in $Mg{\left(OH\right)}_{2}\;$ content, but the CA content had little effect on the LOI of CR vulcanizate, possibly due to the small amount of CA. Comparing CA-1-10 and CA1-10-20, it can be seen that 20 wt% carbon black slightly reduced the LOI of CR vulcanizate.

## 4. Conclusions

Designing and fabricating CR vulcanizates that simultaneously possess exceptional tensile strength, notable elongation at breaks, and remarkable flame-retardant properties present a formidable undertaking. Inspired by nature material manufacture, CR vulcanizates have been prepared by synchronously introducing sacrificial bond (disulfide) crosslinking networks into the chemically crosslinked network. Under the catalysis of $Mg{\left(OH\right)}_{2}$, the dynamic bond crosslinking network is formed by the nucleophilic substitution of the amino groups of CA or DTA with the allylic chlorine groups of CR, while the covalently crosslinked network is synchronously formed through two nucleophilic substitution reactions between $Mg{\left(OH\right)}_{2}\;$ and CR. CA-0.5 vulcanizate with a dual crosslinking network exhibits excellent mechanical performance, with the tensile strength and elongation at breaks of CA-0.5 being 1.41 times and 1.17 higher than those of CR-0 with covalently crosslinked networks, respectively. The disulfide bonds act as sacrificial bonds, which undergo preferential rupture before the covalent network, leading to energy dissipation and facilitating the orientation of rubber chains. This characteristic contributes to the exceptional mechanical properties exhibited by CA-0.5. Due to the decrease in the crosslinking density of DTA-0.5 caused by side reactions, the mechanical properties of DTA-0.5 vulcanizate with double networks are inferior to those of CR-0 vulcanizate with covalently crosslinked networks. Moreover, the addition of $Mg{\left(OH\right)}_{2}\;$ significantly improves the flame retardancy of CR vulcanizates, with the LOI of CA-1-20 being 1.14 times higher than that of CR-0.

## Figures and Tables

**Table 1:** Blend designations and compositions in phr.

Ingredient (phr)	Sample ID													
	CR-0	CR-1	CR-2	CA-0.5	CA-1	CA-2	CA-1-x	CA1-10-20	DTA-0.5	DTA-1	DTA-2	DTA-3	DTA1-x	DTA1-10-20
CR	100	100	100	100	100	100	100	100	100	100	100	100	100	100
$Mg{\left(OH\right)}_{2}$	0	10	0	10	10	10	x	10	10	10	10	10	x	10
$Al{\left(OH\right)}_{3}$	0	0	10	0	0	0	0	0	0	0	0	0	0	0
CA	0	0	0	0.5	1	2	1	1	0	0	0	0	1	1
DTA	0	0	0	0	0	0	0	0	0.5	1	2	3	0	0
ZnO	5	0	0	0	0	0	0	0	0	0	0	0	0	0
CB	0	0	0	0	0	0	0	20	0	0	0	0	0	20
antioxidant 1010	1	1	1	1	1	1	1	1	1	1	1	1	1	1

x: refers to the addition of different masses of $Mg{\left(OH\right)}_{2}$ to the CR mixture.

## Data Availability

Data is unavailable due to privacy or ethical restrictions.
